# Keil’s Medical, Pharmaceutical, and Dental Directory

**Published:** 1897-09

**Authors:** 


### "Keil's Medical, Pharmaceutical, and Dental Directory. Net price, bound in cloth, $3.00; in leather, $4.00. Geo. Keil, Editor and Publisher, 1715 Willington St., Phila., Pa.

George Keil, Philadelphia, announces the early publication (Fifth Bi-Yearly Edition) of *Keil's Medical, Pharmaceutical, and Dental Register-Directory and Intelligencer* for Pennsylvania, New York, New Jersey, Maryland, Delaware, and District of Columbia. Its list of national colleges, State hospitals, homes, dispensaries, societies, laws, and post-office addresses of physicians, druggists, and dentists, school of graduation and year, all the latest laws in these States, will be complete as to date of issue, as a personal canvass will be made from data. It is the only directory published for above-named States, registering graduates of all schools, physicians, druggists, and dentists, and imparting all information needed by the professions mentioned in their daily practice. No effort will be spared to make the *Directory* complete and the information accurate and reliable in the minutest detail belonging to the domain of medical, pharmaceutical, and dental professions. An experience of over thirty years is a sufficient guarantee that all subjects will be properly treated in *Keil's Directory*. The names in large cities, in addition to being in alphabetical order, will be numerically arranged by streets. These features will no doubt be appreciated.

